# Utilization of HIV testing services among pregnant mothers in low income primary care settings in northern Ethiopia: a cross sectional study

**DOI:** 10.1186/s12884-017-1389-2

**Published:** 2017-06-24

**Authors:** Yihun Mulugeta Alemu, Fentie Ambaw, Annelies Wilder-Smith

**Affiliations:** 1 Institute of Public Health, Heidelberg University, Heidelberg, Germany; 2 School of Public Health, College of Medicine and Health Science, Bahir Dar University, Bahir Dar, Ethiopia

**Keywords:** Utilization, HIV testing, Pregnant mothers

## Abstract

**Background:**

HIV testing of women in child bearing age is an entry point for preventing mother-to-child transmission of HIV (MTCT). This study aims to identify the proportion of women tested for HIV and to determine factors associated with utilization of HIV testing services among pregnant mothers in primary care settings in northern Ethiopia.

**Methods:**

A cross sectional study was conducted in 416 pregnant women from four primary care centers between October 2, 2012 and May 31, 2013 in East Gojjam, Ethiopia.

**Results:**

The proportion of mothers who tested for HIV was 277(67%). Among mothers who were not tested for HIV, lack of HIV risk perception (*n* = 68, 49%) was a major self-reported barrier for HIV testing. A multivariable logistic regression analysis showed that those pregnant women who had comprehensive knowledge about MTCT had an Adjusted Odd Ratio (AOR) of 3.73 (95% CI: 1.56, 8.94), having comprehensive knowledge on prevention of mother to child transmission (PMTCT) of HIV an AOR of 2.56 (95% CI: 1.26, 5.19), and a favorable attitude towards persons living with HIV an AOR of 2.42 (95%CI, 1.20, 4.86) were more likely to be tested for HIV.

**Conclusion:**

One third of pregnant women had never been tested for HIV until the time of the study. Efforts should be made to improve mother’s knowledge about MTCT and PMTCT to increase uptake of HIV testing. Enhancing mother’s HIV risk perception to scale up HIV testing in resource limited setting is highly recommended.

**Electronic supplementary material:**

The online version of this article (doi:10.1186/s12884-017-1389-2) contains supplementary material, which is available to authorized users.

## Background

HIV continues to pose a major problem globally. More than 90% of new global pediatric HIV infections are in sub-Saharan Africa [1]. According to the HIV/AIDS estimates of the Central Statistical Agency of Ethiopia in 2014, 3886 children in the country acquire HIV infection every year [2]. Mother to child transmission (MTCT) is a major source for pediatric HIV infection. In the absence of any preventive measures, the risk of a baby acquiring the virus from an infected mother ranges from 15 to 45%, which occurs during pregnancy, childbirth or breast feeding [3].

HIV testing of women of child bearing age is an entry point for PMTCT (prevention of mother to child transmission). It is one of the primary prevention strategies for MTCT. HIV testing is an effective intervention to increase PMTCT [4]. However a significant number of women in HIV epidemic regions have not been tested for HIV [5]. Utilization of HIV testing services in sub-Saharan Africa is low; even where such services are available, women are not receiving the full benefits [1].

Previous studies have identified socio-demographic status [6–13], knowledge of MTCT& PMTCT [14–16] and attitude towards HIV/AIDS [17–19] as factors that determine uptake of HIV testing. However, the relative contribution of these factors to HIV testing varies from study to study and from population to population. Studies conducted in Ethiopia on HIV testing of pregnant mothers were mainly on acceptance and refusal of the test offered by the counselors at PMTCT sites during antenatal care [20–22]. Studies that identify proportions and factors associated with actual HIV testing among pregnant mothers are scarce. This study will contribute to identifying obstacles to take up HIV taking in pregnant women in order to develop a more comprehensive & multidisciplinary approach to scale up HIV testing services in resource limited settings. We aim to identify the proportion of and factors for HIV testing among pregnant women in Ethiopia.

## Methods

### Study design and study settings

We conducted a cross sectional study in health institutions in Amhara region from October 2, 2012 to May 31, 2013. In Amhara regional state, there are three metropolitan cities and ten zones. East Gojjam is one of the ten zones and consists of 15 districts (werda). Sixty nine primary health care centers provide both antenatal care and PMTCT services in the 15 districts. A total 588,633 women of reproductive age (15–49 years) live in the area [23].

### Sample size determination

Sample size was determined using a single population proportion estimation formula and calculated using Epi-info 7 with 50% proportion, 5% of absolute precision, 95% confidence interval and non-response rate of 10%. The calculated overall sample size was 422.

### Sampling procedures

Four primary health care centers from East Gojjam Zone were randomly selected and included in the study. Primary health care centers from Motta, Gendowin, Bitchena and Debrewerk were identified. Pregnant women attending their first antenatal care visit for a current pregnancy at any of those four health institutions was recruited. We employed proportional allocation of subjects to each facility per size, systematic random sampling with sampling interval of three was used to select pregnant women from each health institution.

### Data collection and analysis

Questionnaires were first prepared in English, translated to Amharic followed by back translation and pretested for consistency and ease of understanding. Data were collected from pregnant women during their first antenatal care visit. Trained nurses conducted face to face interviews with study participants by using questionnaires of Amharic language. Data were collected to assess socio-demographic variables (age, education, residence and monthly expenditure), knowledge and attitude variables (knowledge of MTCT & PMTCT and specific attitude to HIV positive living).

Data were entered into Epi info 7 and analysis was done with STATA 12. Frequencies and proportions were used to describe the study subjects in relation to the studied variables. Logistic regression model was used to examine the relation between explanatory variables and HIV testing. Bi-variable logistic regression model were fitted for all explanatory variables. Odds ratio with its 95% confidence interval and *p*-value were used to measure strength of association and to identify statistical significance of results. Identification of confounding variables by using logistic regression model was used. All explanatory variables that were fitted in the bi-variable model were fitted to a multivariable logistic regression model. The clustered nature of the data was taking into account in the analysis; however, multilevel logistic regression model was not fitted. The ICC (intra-class correlation coefficient) estimate was 0.038, indicating that only 3.8% of the variation is due to difference between health institutions. Most of the variations (96.2%) are explained by the lower level measures (pregnant women). As ICC was less than 5–10%, hierarchical modeling is not required.

### Operational definitions

**Comprehensive knowledge of MTCT:** pregnant women were classified as knowledgeable if they knew about mother to child transmission of HIV and were able to list at least two means of transmission (during pregnancy, delivery or breast feeding).

**Comprehensive knowledge of PMTCT:** pregnant woman classified as knowledgeable if the woman knew about PMTCT and method of PMTCT (antiretroviral therapy).

**Favorable attitude towards persons living with HIV:** defined as a woman who perceived that her husband or/and other family member/s will care for her, if her HIV test result is positive.

## Result

### Socio-demographic variables and proportion of HIV testing

Out of the 422 eligible participants, 416 consented to be enrolled into the study; 178 women in Motta hospital, 73 in Debre Werke health center, 77 in Bichena health center, and 88 in Gindwyen health center were recruited. The overall response rate was 98.6% and the mean age was 28.2 years (SD = 6.15 yrs). Table 1 presents the frequency distribution of socio-demographic variables among HIV tested and non-tested subjects; 55% (*n* = 229) of the subjects were from rural areas. Majority of them (53.6%) did not have any formal-education. The proportion of pregnant women who tested for HIV at least once among the study group was 277(67%).

**Table 1:** Socio-demographic characteristics of HIV tested and not-tested pregnant women attending antenatal care in East Gojjam, northern Ethiopia, 2013

Variable	Not-tested n (%)	Tested n (%)	Total (%)
Age			
16–24	24(17)	98(35)	122(29)
25–29	34(24)	99(36)	133(32)
30–34	30(22)	57(21)	87(21)
≥ 35	51(37)	23(8)	74(18)
Residence			
Rural	113(50)	116(51)	229(55)
Urban	26(14)	161(86)	187(45)
Education			
No formal education	111(80)	112(40)	223(54)
Primary education	11(8)	27(10)	38(10)
Secondary above	17(12)	138(50)	155(37)
Monthly expenditure			
< 30 USD	128(92)	172(62)	300(72)
≥ 30 USD	11(8)	105(38)	116(28)
Parity			
0	25(18)	95(79)	120(29)
1–4	67(48)	150(69)	217(52)
≥ 5	47(34)	32(42)	79(19)

### Knowledge and attitude related to HIV testing

Table 2 shows the frequency distribution of knowledge and attitude variables among HIV tested and non-tested. From 416 mothers, 74% (tested = 76%, non-tested = 24%) of the study population had heard of mother to child transmission of HIV. Out of these, 70% (tested = 79%, non-tested = 21.0%) knew of mother to child transmission of HIV during pregnancy; 48% (tested = 86%, non-tested = 14%) knew of mother to child transmission of HIV during delivery and 70% (tested = 81% non-tested = 19%) knew of mother to child transmission of HIV during breast feeding. Similarly, among 307 mothers, 88% (tested = 82%, non-tested = 18%) heard of PMTCT. In regard to specific attitude towards HIV positive living among 416 pregnant women, 38% (tested = 85%, non-tested = 15%) perceived their husband or/and other family member/s will care for them, if their HIV test results are positive.

**Table 2:** Knowledge and attitude related to HIV testing among pregnant women attending antenatal care in health facilities of East Gojjam, northern Ethiopia 2013(*n* = 416)

Variables	Non-tested n (%)	Tested n (%)	Total n (%)
Knowledge of MTCT and PMTCT			
Heard mother to child transmission			
Yes	72(24)	235(76)	307(74)
No	67(62)	42(38)	109(26)
Transmission can occur during pregnancy (*n* = 307)			
Yes	45(21)	169(79)	214(70)
No	27(29)	66(71)	93(30)
Transmission can occur during delivery(*n* = 307)			
Yes	21(14)	125(86)	146(48)
No	51(32)	110(68)	161(52)
Transmission can occur during breast feeding (*n* = 307)			
Yes	41(19)	172(81)	213(70)
No	31(33)	63(67)	94(30)
Heard PMTCT (*n* = 307)			
Yes	49(18)	222(82)	271(88)
No	23(64)	13(36)	36(12)
Specific attitude to HIV positive living (*n* = 416)			
What will be the reactions if your HIV test result is positive?			
No one believe the test result			
Yes	54(32)	116(68)	170(41)
No	85(35)	161(65)	246(59)
I will be thrown out of home/outcast			
Yes	32(39)	50(61)	82(20)
No	107(32)	227(68)	334(80)
I will be physically violated/abused			
Yes	10(27)	27(73)	37(9)
No	129(34)	250(66)	379(91)
My family will start to care for me			
Yes	24(15)	135(85)	159(38)
No	115(45)	142(55)	257(62)

### Self-reported reasons of mother’s not undertook HIV testing

Table 3 shows self-reported reasons of mothers not taking up HIV testing. The most frequent reason that pregnant women were not tested for HIV was due to the lack of HIV risk perception (49%) followed by the absence of nearby HIV testing center (26%). Other reasons included lack of perceived benefit of HIV testing (22%) and perceived lack of confidentiality in HIV testing centers (7%).

**Table 3:** Self-reported reasons for not-testing for HIV among pregnant women attending antenatal care in health facilities of East Gojjam, northern Ethiopia 2013

Reasons for not HIV testing *n* = 139	
What is/are your reasons not tested for HIV?	
I have no risk for HIV	n (%)
Yes	68(49)
No	71(51)
Lack of HIV testing center	
Yes	36(26)
No	103(74)
Knowing HIV status has no benefit	
Yes	30(22)
No	109(78)
Not aware of HIV testing service	
Yes	26(19)
No	113(81)
Perceived lack of confidentiality to the HIV testing centers	
Yes	10(8)
No	129(92)

### Bi-variable analysis of factors associated with HIV testing

Table 4 presents the relation between explanatory variables and HIV testing. The bi-variable analysis shows that women with the age of 16–24 years had 9 times higher odds (crude odd ratio [COR] = 9, 95% CI: 4.65, 17.59) of being tested for HIV compared to pregnant women who were older (≥35 years). Women who had secondary education and above were (COR = 8.04, 95% CI: 4.55, 14.10) more likely to be tested for HIV compared with women who did not attend formal education. Mothers from urban areas were (COR = 6.03, 95% CI: 3.70, 9.83) more likely to be tested for HIV compared with mothers from rural areas. Mothers whose monthly expenditure was ≥30 USD (COR = 7.10, 95%CI: 3.66, 13.76) were more likely to be tested for HIV compared with mothers whose monthly expenditure was <30 USD. Nulliparous women (primigravidae) were (COR = 5.58, 95%CI: 2.97, 10.47) more likely to be tested for HIV compared with pregnant women who had five or more children.

**Table 4:** Factors associated with HIV testing among pregnant women attending antenatal in East Gojjam, northern Ethiopia, 2013

Variable	Not-tested n (%)	Tested n (%)	COR	95%CI	*P*-value
Age					
16–24	24(17)	98(35)	9.05	4.65,17.59	<0.0001
25–29	34(24)	99(36)	6.65	3.54,12.49	<0.0001
30–34	30(22)	57(21)	4.21	2.17, 8.16	<0.0001
≥ 35	51(37)	23(8)	1		
Education					
No formal education	111(80)	112(40)	1		
Primary education	11(8)	27(10)	2.43	1.15,5.14	0.020
Secondary education or above	17(12)	138(50)	8.04	4.55,14.1	<0.0001
Residence					
Rural	113(49)	116(51)	6.03	3.70, 9.83	< 0.0001
Urban	26(14)	161(86)	1		
Monthly expenditure					
< 30 USD	128(43)	172(57)	1		
≥30 USD	11(10)	105(90)	7.10	3.66,13.76	<0.0001
Parity					
0	25(18)	95(34)	5.58	2.97, 10.47	<0.0001
1–4	67(48)	150(54)	3.28	1.92, 5.60	<0.0001
≥ 5	47(34)	32(12)	1		
Comprehensive knowledge of MTCT					
Yes	10(9)	155(56)	16.38	8.25, 32.53	<0.0001
No	129(93)	122(44)	1		
Comprehensive Knowledge of PMTCT					
Yes	26(19%)	190(69%)	9.49	5.77,15.58	<0.0001
No	113(81%)	87(31%)	1		
Favored attitude to HIV positive living					
Yes	24(17%)	135(48%)	4.55	2.76, 7.50	<0.0001
No	115(83%)	142(51%)	1		

Study subjects who had comprehensive knowledge of mother to child transmission of HIV were (COR = 16.03, 95% CI: 8.25, 32.53) more likely to be tested for HIV. Respondents who had comprehensive knowledge of PMTCT were (COR = 9.49, 95% CI: 5.77, 15.58) more likely to be tested for HIV. Women who had favored attitude to HIV positive living were (COR = 4.55, 95%CI, 2.76, 7.50) more likely to be tested for HIV.

### Multivariable analysis of factors independently associated with HIV testing

Table 5 shows independent factors associated with HIV testing. After controlling for possible confounders using a multivariable logistic regression model, women age 16–24 years were more likely to be tested for HIV compared with older pregnant women (≥35 years) (AOR = 7.9, 95% CI: 3.19, 19.55). Women who had secondary education and above were more likely to be tested for HIV compared with women who did not have formal education (AOR = 3.49, 95% CI: 1.56, 7.77). Mothers from urban areas were more likely to be tested for HIV compared with women from rural areas (AOR = 3.42, 95% CI: 1.82, 6.46). Mothers whose monthly expenditure was ≥30 USD were more likely to be tested for HIV compared with mothers whose monthly expenditure was <30 USD (AOR = 4.06, 95%CI: 1.66, 9.93). Nulliparous women were more likely to be tested for HIV compared with pregnant women who had five or more children (AOR = 4.34, 95%CI: 1.61, 11.68).

**Table 5:** Factors independently associated with HIV testing among pregnant women attending antenatal in East Gojjam, northern Ethiopia, 2013

Variable	COR(95%CI)	*P*-value	AOR(95%CI)	*P*-value
Age group				
16–24	9.05 (4.65,17.59)	<0.0001	7.90(3.19,19.55)	< 0.0001
25–29	6.65 (3.54,12.49)	<0.0001	4.95(2.06,11.88)	< 0.0001
30–34	4.21 (2.17, 8.16)	<0.0001	3.31(1.27, 8.60)	0.014
≥ 35	1		1	
Education				
No formal education	1		1	
Primary education	2.43 (1.15, 5.14)	0.017	0.80(0.28, 2.32)	0.690
Secondary above	8.04 (4.55,14.19)	< 0.0001	3.49(1.56, 7.77)	0.002
Residence				
Urban	6.03(3.70, 9.83)	< 0.0001	3.42(1.82,6.46)	<0.0001
Rural	1			
Monthly expenditure				
≥ 30 USD	1			
< 30 USD	7.10(3.66,13.76)	<0.0001	4.06(1.66,9.93)	0.002
Parity				
0	5.58 (2.97,10.47)	<0.0001	4.34(1.61,11.68)	0.004
1–4	3.28 (1.92, 5.609	<0.0001	4.70(1.94,11.36)	0.001
≥ 5	1			
Comprehensive knowledge of MTCT				
Yes	16.38(8.25,32.53)	<0.0001	3.73(1.56,8.94)	0.003
No	1			
Comprehensive Knowledge of PMTCT				
Yes	9.49 (5.77,15.58)	<0.0001	2.56(1.26, 5.19)	0.009
No	1			
Favored attitude to HIV positive living				
Yes	4.55(2.76, 7.50)	<0.0001	2.42(1.20,4.86)	0.003
No	1			

Subjects who had comprehensive knowledge of mother to child transmission of HIV were more likely to be tested for HIV (AOR = 3.73, 95% CI: 1.56, 8.94), and so were those who had comprehensive knowledge of PMTCT (AOR = 2.56, 95% CI: 1.26, 5.19). Women who had a favorable attitude to persons living with HIV more likely to be tested for HIV (AOR = 2.42, 95%CI, 1.20, 4.86).

## Discussion

In this study 67% of pregnant women were tested for HIV. It was lower than studies conducted in Vietnam (90%) and Cambodia (76%) [24, 25]. This could be due to differences of in knowledge and attitude towards HIV/ADIS, MTCT or PMTCT.

In our study women of older age were less likely to be tested for HIV. In contrast a study conducted in Cameron showed that pregnant women between the age range of 25 and 30 years were more likely to be tested for HIV compared with pregnant women age less than 20 years. On the other hand, a study conducted in Georgia showed that the age of the mother was not a determinant of opting for HIV testing [26].

Our study also showed that pregnant women who attended secondary education or above were more likely to be tested for HIV compared with pregnant women who did not attend formal education, consistent with previous studies in Nigeria and Tanzania that showed an association between higher educational level and HIV testing [7, 9]. However other studies level of education was a statistically significant factor for HIV testing [25, 26]. This could be due to variations in what is taught at schools.

The findings of this study showed that women from urban areas were more likely to be tested for HIV compared with women from rural areas and this is consistent with other studies [16, 17, 25]. This could be due to lower access of HIV testing centre in women from rural areas. Women who lived closer to the hospital were more likely to be tested for HIV [10].

A study showed that lower income was associated with a lower likelihood of pregnant women getting counseling for HIV testing [11]. Similarly our study indicated that women whose monthly expenditure was ≥30 USD were more likely to be tested for HIV compared with women whose monthly expenditure was <30 USD. HIV testing service in Ethiopia is free of charge for study participant as well as the general population. The observed association between monthly expenditure and HIV testing could be related to the capacity to afford transport and access to information such as having radio and television. Distance and transport costs were barriers to access PMTCT services [12].

HIV testing among women was negatively associated with parity. Compared to nulliparous women, parous women were more likely to refuse HIV testing after counseling [6, 13]. In this study, pregnant women who have not had any children were more likely to be tested for HIV compared with multiparous pregnant women who had five or more children.

Pregnant women attending antenatal care who had previous knowledge about prevention of mother-to-child transmission of HIV were more likely to be tested for HIV [14, 15]. In this study subjects who had comprehensive knowledge of mother to child transmission of HIV were more likely to be tested for HIV. Pregnant women with a high PMTCT knowledge score and knowing someone who receives antiretroviral therapy for PMTCT, were more likely to be tested for HIV [16]. Similarly in our findings respondents who had comprehensive knowledge of PMTCT were more likely to be tested for HIV. This could be due to higher perceived benefit of HIV testing among women who had better knowledge of MTCT and PMTCT. Studies have identified lack of HIV risk perception, perception of poor health care support, worry about husband’s disapproval, fear of knowing their HIV status, need to have their partner consent, stigma and poor perceived quality of post-test counseling for positive test result as some of the reasons women were tested for HIV [16–19]. Some of the potential gaps for HIV testing for pregnant women are lack of knowledge, stigma and discrimination. According to guidelines on the prevention of mother-to-child transmission of HIV in Ethiopia, male partner involvement, community based mother’s support, involving PLWHIV (people live with HIV) in campaigns to reduce stigma and discrimination are strategies to increase the uptake of HIV testing and PMTCT service as a whole [3]. Efforts to increase knowledge on HIV testing amongst mothers, to decrease stigma of HIV, and to increase partner support have been in place after our study was conducted. These efforts could make HIV testing better today in our study population. However, studies identified that only 20% of male partners are involved PMTCT of similar settings in northern Ethiopia [27, 28].

In this study mothers who lack of HIV risk perception were less likely to be tested for HIV. On the other hand, mothers who had a favorable attitude towards HIV positive living were more likely to be tested for HIV. Many women were not tested for HIV because of perceived low risk of HIV. Among Ethiopian women HIV prevalence in 2013 was 1.7% [2].

### Limitation

Since our study was a cross sectional design, cause-effect relationship could not be established. This study was limited to health institutions and therefore it cannot be generalized to pregnant women who are not able to attend antenatal care in the health care institutions.

## Conclusion

One third of pregnant women had never been tested for HIV until the time of the study. Implementing information, education and behavioral change communication on HIV positive living, MTCT and PMTCT are very useful to scale up HIV testing. Enhancing mother’s HIV risk perception in resource limited setting is highly recommended (Additional file 1).

## Additional file

Additional file 1: A copy of study questionnaire. (DOCX 24 kb)

## Abbreviations

AOR: Adjusted Odd Ratio
COR: Crude odd ratio
ICC: Intra-class correlation coefficient
MTCT: Mother-to-child transmission of HIV
PMTCT: Prevention of mother to child transmission

## References

[1] USAID (2013). Report on the global AIDS epidemic: USAID.

[2] Central Statistical Agency (2014) HIV/AIDS Estimates and Projections in Ethiopia, 2011–2016. Central Stastistical Agenency, 2014.

[3] Federal Ministry of Health Ethiopia (2011) Guidelines for prevention of mother to child transmission of HIV in Ethiopia. Ministry of Health, 2011.

[4] USAID (2001). Counselling and voluntary HIV testing for pregnant women in high HIV prevalence countries.

[5] Gourlay A, Wringe A, Todd J, Cawley C, Michael D, Machemba R (2015). Factors associated with uptake of services to prevent mother-to-child transmission of HIV in a community cohort in rural Tanzania. Sex Transm Infect.

[6] Mahmoud MM, Nasr AM, Gassmelseed DE, Abdalelhafiz MA, Elsheikh MA, Adam I (2007). Knowledge and attitude toward HIV voluntary counseling and testing services among pregnant women attending an antenatal clinic in Sudan. J Med Virol.

[7] Iliyasu Z, Kabir M, Galadanci H, Abubakar I, Aliyu M (2005). Awareness and attitude of antenatal clients towards HIV voluntary counselling and testing in Aminu Kano teaching hospital, Kano, Nigeria. Niger J Med.

[8] Westheimer EF, Urassa W, Msamanga G, Baylin A, Wei R, Aboud S (2004). Acceptance of HIV testing among pregnant women in Dar-es-salaam. Tanzania. J Acquir Immune Defic Syndr..

[9] Rosa H, Goldani MZ, Scanlon T, da Silva AA, Giugliani EJ, Agranonik M (2006). Barriers for HIV testing during pregnancy in southern Brazil. Rev Saude Publica.

[10] Nguyen LT, Christoffersen SV, Rasch V (2010). Uptake of prenatal HIV testing in Hai Phong Province, Vietnam. Asia Pac J Public Health.

[11] Goldani MZ, Giugliani ER, Scanlon T, Rosa H, Castilhos K, Feldens L (2003). Voluntary HIV counseling and testing during prenatal care in Brazil. Rev Saude Publica.

[12] O'Gorman DA, Nyirenda LJ, Theobald SJ (2010). Prevention of mother-to-child transmission of HIV infection: views and perceptions about swallowing nevirapine in rural Lilongwe, Malawi. BMC Public Health.

[13] Kongnyuy EJ, Mbu ER, Mbopi-Keou FX, Fomulu N, Nana PN, Tebeu PM (2009). Acceptability of intrapartum HIV counselling and testing in Cameroon. BMC Pregnancy Childbirth..

[14] Veloso VG, Portela MC, Vasconcellos MT, Matzenbacher LA, Vasconcelos AL, Grinsztejn B (2008). HIV testing among pregnant women in Brazil: rates and predictors. Rev Saude Publica.

[15] Kominami M, Kawata K, Ali M, Meena H, Ushijima H (2007). Factors determining prenatal HIV testing for prevention of mother to child transmission in Dar Es Salaam, Tanzania. Pediatr Int.

[16] Creek T, Ntumy R, Mazhre J, Smani L, Mooith M, Han G (2009). Factors associated with low early uptake of a national program to prevent mother to child transmission of HIV (PMTCT): results of a survey of mothers and providers, Botswana, 2003. AIDS Behav.

[17] Karamagi C, Tumwine J, Tylleskar T, Heggenhougen K (2006). Antenatal HIV testing in rural eastern Uganda in 2003: incomplete rollout of the prevention of mother-to-child transmission of HIV programme?. BMC Int Health Hum Rights.

[18] Perez F, Zvandaziva C, Engelsmann B, Dabis F (2006). Acceptability of routine HIV testing ("opt-out") in antenatal services in two rural districts of Zimbabwe. J Acquir Immune Defic Syndr.

[19] Dinh TH, Detels R, Nguyen MA (2005). Factors associated with declining HIV testing and failure to return for results among pregnant women in Vietnam. AIDS.

[20] Abebaw D, Amare D, Mulumebet A (2009). Determinants of acceptance of voluntary HIV testing among antenatal clinic attendees at Dil Chora hospital, Dire Dawa, East Ethiopia. Ethiop J Health Dev.

[21] Worku G, Enquselassie F (2007). Factors determining acceptance of voluntary HIV counseling and testing among pregnant women attending antenatal clinic at army hospitals in Addis Ababa. Ethiop Med J.

[22] Malaju MT, Alene GD (2012). Assessment of utilization of provider-initiated HIV testing and counseling as an intervention for prevention of mother to child transmission of HIV and associated factors among pregnant women in Gondar town, north West Ethiopia. BMC Public Health.

[23] Amhara Regional Health Bureau (2013). Amhara health profile 2013.

[24] Sasaki Y, Ali M, Sathiarany V, Kanal K, Kakimoto K (2010). Prevalence and barriers to HIV testing among mothers at a tertiary care hospital in Phnom Penh, Cambodia. Barriers to HIV testing in Phnom Penh, Cambodia. BMC Public Health.

[25] Hạnh NT, Gammeltoft TM, Rasch V (2011). Number and timing of antenatal HIV testing: evidence from a community-based study in northern Vietnam. BMC Public Health.

[26] Maia B, Maia K, George K, Patrice T, Annabel DdL, François D, Joanna O-G. Factors Associated with HIV Testing History among Pregnant Women and Their Partners in Georgia: The ANRS 12127 Prenahtest Trial. AIDS Res Treat 2014;6. 10.1155/2014/356090 PMC4195251 25328692

[27] Amano A, Musa A (2016). Male involvement in PMTCT and associated factors among men whom their wives had ANC visit 12 months prior to the study in Gondar town, north west Ethiopia, December, 2014. Pan African Med J.

[28] Haile F, Brhan Y (2014). Male partner involvements in PMTCT: a cross sectional study, Mekelle, northern Ethiopia. BMC Pregnancy Childbirth.

