# Supplementary material for: Utilization of HIV testing services among pregnant mothers in low income primary care settings in northern Ethiopia: a cross sectional study
Source: BMC Pregnancy Childbirth. 2017 Jun 24;17:199. doi: 10.1186/s12884-017-1389-2 (PMC5483315; doi:10.1186/s12884-017-1389-2)
Supplement: Additional file 1: — A copy of study questionnaire. (DOCX 24 kb) [file 12884_2017_1389_MOESM1_ESM.docx]

**Annex I. Questionnaire**

General information

|  | Question items | Response | Code | Skip |
| --- | --- | --- | --- | --- |
| 01 | Health institution | Motta  Gindewine  Bichna  Debrewerke | 1  2  3  4 |  |
| 02 | Date of interview | ---date-----month ---------year |  |  |

Section one: Socio demographic characteristics and Background information

|  | Question items | Response | Code | Skip to |
| --- | --- | --- | --- | --- |
| 03 | Residence address | Urban  Rural | 1  2 |  |
| 04 | Age | in year |  |  |
| 05 | Number of children |  |  |  |
| 07 | Educational status | Not attend formal education  Attended elementary school/grade 1-8  Attended high school /grade 9-12  Attended college /University | 1  2  3  4 |  |

| 8 | Occupation | Unemployed  Unemployed | 1  2 |  |
| --- | --- | --- | --- | --- |
| 9 | Household expenditure per month | Less than 30 USD  Greater than 30 USD | 1  2 |  |

Section two: knowledge and belief of HIV transmission, MTCT, PMTCT

| No | Items | Response | Code | Skip |
| --- | --- | --- | --- | --- |
| 1 | Know the mode of transmission of HIV | Sexual intercourse | 1 |  |
|  |  | Blood and blood product | 2 |  |
|  |  | MTCT | 3 |  |
|  |  | Injection and blades | 4 |  |
|  |  | I don’t know | 5 |  |
|  |  | Others specify |  |  |
| 2 | Know method/s to prevent being infected with HIV | Abstinence | 1 |  |
|  |  | Faithful | 2 |  |
|  |  | Use condom | 3 |  |
|  |  | I don’t know | 4 |  |
|  |  | Others specify |  |  |
| 3 | Ever heard mother to child transmission? | Yes | 1 |  |
|  |  | No |  | 5 |
| 4 | Possible mother to child transmission | During pregnancy | 1 |  |
|  |  | During delivery | 2 |  |
|  |  | During breast feeding | 3 |  |
|  |  | Others specify |  |  |
| 5 | Ever heard mother to child transmission can be prevented? | Yes | 1 |  |
|  |  | No | 2 | 7 |
| 6 | Method to prevent mother to child  transmission of HIV | Chemotherapy (ARV ) | 1 |  |
|  |  | Vaccination | 2 |  |
|  |  | Traditional therapy | 3 |  |
|  |  | Others (specify ) |  |  |

**Section: Three PMTCT service and antenatal care**

| 1 | Ever tested for HIV testing before? | Yes | 1 |  |
| --- | --- | --- | --- | --- |
|  |  | No | 2 | 3 |
| 2 | If Not, why | Not aware of VCT | 1 |  |
|  |  | Lack of VCT centre access | 2 |  |
|  |  | I have no risk HV | 3 |  |
|  |  | Knowing sero-status no benefit | 4 |  |
|  |  | My husband not allow | 5 |  |
|  |  | Lack of confidentiality to test result | 6 |  |
| 3 | Would you think, you have risk of acquiring HIV? | Yes | 1 |  |
|  |  | No | 2 |  |
| 4 | If you result turns out to be positive, what would be the likely reaction to husband or relatives? | No one will believe the results | 1 |  |
|  |  | I will be thrown out of home | 2 |  |
|  |  | I will be physically violated/ abused | 3 |  |
|  |  | He/ they will start to care for | 4 |  |
